# Histidine-Mediated pH-Sensitive Regulation of M-Ficolin:GlcNAc Binding Activity in Innate Immunity Examined by Molecular Dynamics Simulations

**DOI:** 10.1371/journal.pone.0019647

**Published:** 2011-05-05

**Authors:** Lifeng Yang, Jing Zhang, Bow Ho, Jeak Ling Ding

**Affiliations:** 1 Computational and Systems Biology, Singapore-MIT Alliance (SMA), Singapore, Singapore; 2 Department of Biological Sciences, National University of Singapore, Singapore, Singapore; 3 NUS Graduate School for Integrative Science and Engineering (NGS), National University of Singapore, Singapore; 4 Department of Microbiology, Yong Loo Lin School of Medicine, Singapore, Singapore; University of Georgia, United States of America

## Abstract

**Background:**

M-ficolin, a pathogen recognition molecule in the innate immune system, binds sugar residues including N-acetyl-D-glucosamine (GlcNAc), which is displayed on invading microbes and on apoptotic cells. The *cis* and *trans* Asp282-Cys283 peptide bond in the M-ficolin, which was found to occur at neutral and acidic pH in crystal structures, has been suggested to represent binding and non-binding activity, respectively. A detailed understanding of the pH-dependent conformational changes in M-ficolin and pH-mediated discrimination mechanism of GlcNAc-binding activity are crucial to both immune-surveillance and clearance of apoptotic cells.

**Methodology/Principal Findings:**

By immunodetection analysis, we found that the pH-sensitive binding of GlcNAc is regulated by a conformational equilibrium between the active and inactive states of M-ficolin. We performed constant pH molecular dynamics (MD) simulation at a series of pH values to explore the pH effect on the *cis-trans* isomerization of the Asp282-Cys283 peptide bond in the M-ficolin fibrinogen-like domain (FBG). Analysis of the hydrogen bond occupancy of wild type FBG compared with three His mutants (H251A, H284A and H297A) corroborates that His284 is indispensible for pH-dependent binding. H251A formed new but weaker hydrogen bonds with GlcNAc. His297, unlike the other two His mutants, is more dependent on the solution pH and also contributes to *cis-trans* isomerization of the Asp282-Cys283 peptide bond in weak basic solution.

**Conclusions/Significance:**

Constant pH MD simulation indicated that the *cis* active isomer of Asp282-Cys283 peptide bond was predominant around neutral pH while the *trans* bond gradually prevailed towards acidic environment. The protonation of His284 was found to be associated with the *trans-to-cis* isomerization of Asp282-Cys283 peptide bond which dominantly regulates the GlcNAc binding. Our MD simulation approach provides an insight into the pH-sensitive proteins and hence, ligand binding activity.

## Introduction

Ficolins are one of the most important groups of pathogen-recognition receptors in the innate immune system [1], [2], [3]. Ficolins are trimeric oligomers, which are linked by disulfide bonds at the N-terminus. The polypeptide chains are comprised of N-terminal collagen-like domain and C-terminal fibrinogen-like (FBG) domain [1], [3], [4], [5], [6]. Among the three characterized human ficolins, the L- and H-ficolins are serum-type proteins while the M-ficolin is mainly detected in and on the immune cells [4], [7], [8], [9], [10], [11], [12], and minimally in the human plasma [13], [14]. The L- and M-ficolins share high homology, with amino acid sequence identity of 80%, whereas the primary structure of H-ficolin is only 48% identical to that of L- and M-ficolins [7], [11]. Binding of ficolins to the specific ligands on the surface of pathogens and antigens activates the lectin complement pathway [2], [15], [16]. Human ficolins recognize pathogen-associated molecular patterns such as lipoteichoic acid [17] and 1,3-β-D-glucan [8]. The ficolins bind to the acetyl groups on microbial surfaces such as N-acetyl-D-glucosamine (GlcNAc), which is a chemical moiety on the pathogen-associated molecular patterns [3], [4], [7], [8], [9], [10], [15], [16], [18]. GlcNAc is also found on apoptotic host cells. Therefore the ability of ficolins to differentiate between the GlcNAc residues exposed on the apoptotic host cells and those on the pathogens, is crucial for self-nonself recognition.

Ficolins also collaborate with C-reactive protein (CRP), which is an acute phase protein known to initiate the complement classical pathway [19], [20]. Interaction between ficolin and CRP forms stable pathogen recognition complexes, which activate the lectin complement pathway [12], [21], [22]. Under local infection-inflammation condition (pH 6.5 and 2 mM Ca^2+^), the M-ficolin is secreted out of the immune cells. At pH 6.5, the binding affinity between both the L-ficolin & CRP and the M-ficolin & CRP were shown to increase significantly compared to their interaction at pH 7.4 [12], [20]. Our recent studies showed that pH 6.5 induced a subtle change in the conformation of the membrane-associated M-ficolin, which strengthened its ternary cross-talk with CRP and G-protein coupled receptor 43, a transmembrane receptor), thus blocking the GlcNAc (ligand) binding site of the M-ficolin [12]. Furthermore, the ligand-binding site on the M-ficolin was proposed to function as a pH-sensitive switch [6], [23], [24], [25].

The crystal structures of the trimeric recognition domains of the three human ficolins on their own, and in complex with different ligands have been reported [6], [23], [26]. Recently, the crystal structure of the mutant M-ficolin FBG, Y271F (referred as Y300F in this paper), has been determined [27]. The ficolin FBG domain contains three subdomains, A, B and P [23]. While the L-ficolin contains three GlcNAc-binding sites in its FBG [26], the M-ficolin contains only one GlcNAc-binding site in the P subdomain, which is close to the Ca^2+^ binding site [6], [25], [26]. At pH 5.6, the GlcNAc binding site is dislocated and the *cis*-Asp282-Cys283 peptide bond near the binding site, which was observed at pH 7.0 in the crystal structure, shows a *trans* conformation, suggesting a pH-sensitive region in the ligand-binding site of the M-ficolin [6], [23]. Analyses of the pH-dependent GlcNAc binding activity of M-ficolin suggested a 2-state conformational equilibrium model having a pK_a_ of 6.2, which may contribute to the binding ability [23]. Tanio et al. [28] performed site-directed mutagenesis study of His residues at pH 8.0 and suggested that His251, His284 and His297 in the P subdomain of M-ficolin are responsible for the pH-dependent activity of M-ficolin. However, the precise contribution of each of the His residues towards the binding of GlcNAc, remains unclear.

In the present study, constant pH Molecular Dynamics (MD) simulations were performed on the wild type (WT) FBG domain of M-ficolin, at each of the pH 4.5, 5.6, 6.0, 6.1, 6.18, 6.2, 6.5, 7.0, and 7.4, to identify the pH-regulated conformational switch in the recognition domain at the atomic level. We found that the *cis-trans* isomerization of the Asp282-Cys283 peptide bond observed at pH 7.0 and 5.6 is consistent with the crystal structure. We also showed that the degree of *cis-trans* isomerization of the Asp282-Cys283 peptide bond varies at different pH. This is consistent with our experimental immunodetection results, which indicated a gradual rise in the interaction between M-ficolin and GlcNAc, achieving a maximum around pH 6.2. The Asp282-Cys283 peptide bond gradually switched to the *cis-*conformation and became the dominant form at pH 6.2, during which maximal binding interaction occurred between the M-ficolin and GlcNAc. The protonation states of His284 were suggested to cause the *cis-trans* isomerization of the Asp282-Cys283 peptide bond in this pH range. Furthermore, we compared the hydrogen bonds of GlcNAc with WT FBG and the His mutants. We found that the mutants displayed different modes of binding GlcNAc, and different abilities in regulating the *cis-trans* isomerization of the Asp282-Cys283 peptide bond. His284 was found to be the crucial residue controlling GlcNAc binding, and His251 enhanced the interaction with the same binding mode as WT FBG, while His297 was more affected in weak basic solution pH.

## Results and Discussion

### Prediction of the pKa

**Figure 1:**
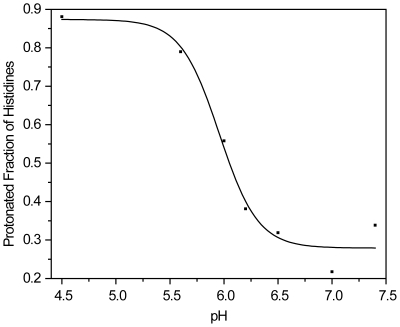
The protonated fraction for M-ficolin FBG Histidines. The calculations were from at least 20 ns simulations at each pH value between 4.5 and 7.4 (midpoint of pKa values also presented in Table 1). The solid line represents best fit titration curve for all His residues with pKa of 6.1.

To demonstrate the conformational changes of M-ficolin FBG at a specified pH, we scanned the pH in proximity, from pH 4.5 to 7.4, titrating with all Asp, Glu, and His residues. The method used to model the pH values was aimed at changing the protonation states of the titrating residues. The interaction with other titratable groups may lead to the magnitude difference between the pH and the predicted pKa in the studied pH range because the residue spent much more time in either the protonated or deprotonated states [29], [30]. We found that the protonation states of His residues changed dominantly and the titration curves of Asp and Glu residues showed less protonation states over this pH range. A valid constant pH MD simulation method should yield pK_a_ predictions consistent with experimental values. The curves showed that the pK_a_ of the His residues is 6.1 (Figure 1). Table 1 summarizes the midpoint pK_a_ prediction of the His residues in M-ficolin FBG, which validates the credibility of the method used in this work [31]. Unfortunately, no experimental pKa values of M-ficolin are available for individual comparison, although Tanio et al. [23], [25], [28] had already used an experimental model to study the GlcNAc binding profile. In these studies, the analysis of the pH-dependent GlcNAc binding activity of M-ficolin had suggested three His residues (His251, His284 and His297) to regulate the activity. Therefore, we investigated the His residues in the P subdomain in greater detail and propose that His284 is the strong candidate contributing to *cis-trans* isomerization of Asp282-Cys283 peptide bond under weak acidic to neutral pH, as described below.

**Table 1:** The midpoint pKa predictions for histidine residues on M-ficolin FBG from constant pH simulations.

pKa (prediction)						pKa^66^ (reference)	
His132	His194	His215	His251	His284	His297	His-δ	His-ε
6.25	6.3	6.05	6.45	6.25	6.1	6.5	7.1

### Structural transition in M-ficolin at pH 6.5 and pH 7.4

**Figure 2:**
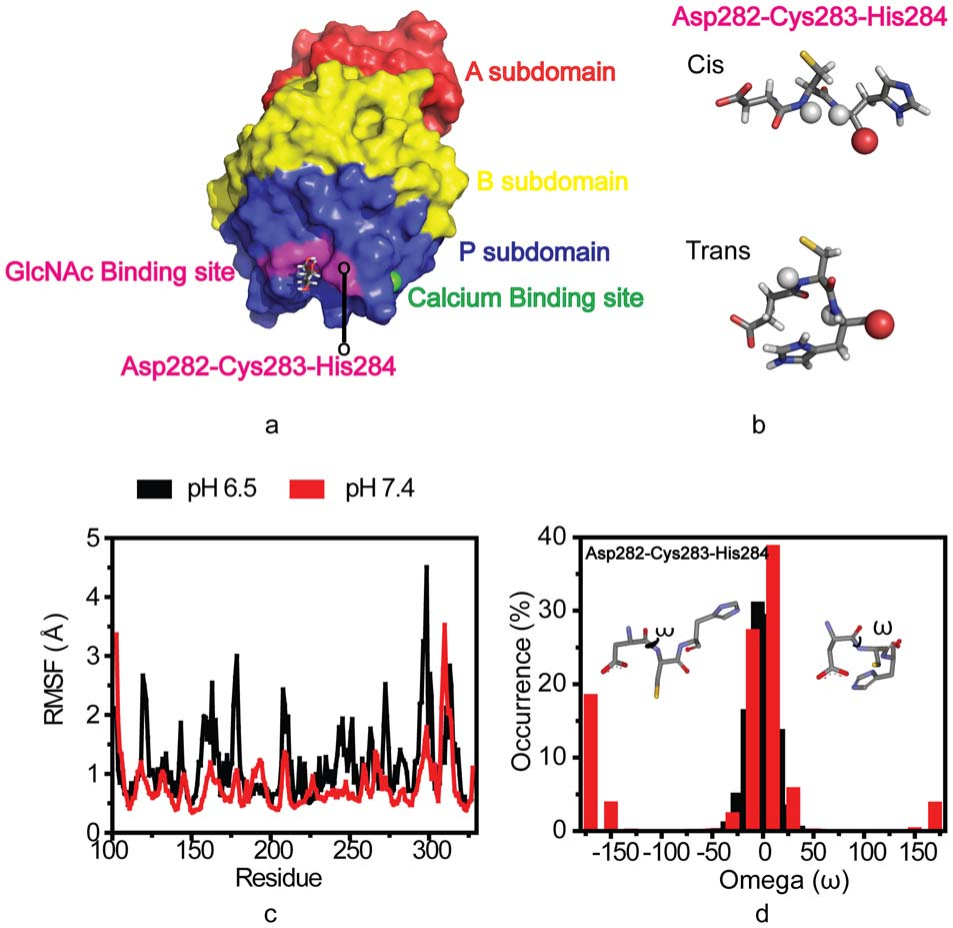
Molecular information on the M-ficolin FBG domain and simulation at pH 6.5 and 7.4. (a) Structure of M-ficolin FBG domain showing the A, B and P subdomains. The calcium and GlcNAc binding sites are annotated. (b) *cis*-*trans* conformation of Asp282-Cys283 peptide bond with the atoms involved in H-bond are shown as spheres. (c) Backbone root mean square deviation (RMSD) from crystal coordinates of residues of FBG P domain. (c) Alpha carbon root mean square fluctuation (RMSF) of residues of M-ficolin FBG domain. The RMSF profile indicates that the RMSD is mainly due to the loop flexibility. (d) The occurrence of dihedral angle (ω) of Asp282-Cys283 peptide bond in whole simulations. Black represents pH 6.5 and red represents pH 7.4 plots individually.

The A, B and P subdomains of the M-ficolin FBG domain and the GlcNAc-binding site, which is close to the Ca^2+^ binding site, are shown in Figure 2a. GlcNAc binding site, stabilized with the hydrophobic pocket and hydrogen bonds by Phe274, Asp282, Cys283, His284, Tyr300, Ala301 and Tyr312, is located in the P subdomain. The tripeptide, Asp282-Cys283-His284, is important to maintain the GlcNAc binding site. The *cis* and *trans* forms of this Asp282-Cys283 peptide were observed at pH 7.0 and 5.6. At pH 5.6, the hydrogen bonds were broken (Figure 2b), indicating the potential disruption of the binding site at different pH during the *cis-trans* isomerization of the Asp282-Cys283 peptide bond. To elucidate the pH-mediated GlcNAc binding activity of M-ficolin, we used constant pH MD to study the structure and dynamics of the GlcNAc binding site in M-ficolin FBG domain.

Compared to traditional MD, constant pH MD is the recently developed method to study how pH affects the protonation status-dependent dynamics [30], [32]. We benefited from the generalized Born (GB) implicit solvation constant pH MD model notwithstanding its moderate increase of computational cost compared to traditional GB MD. The backbone root mean square deviations (RMSD) value was selected as the main parameter since the conformational change was of particular interest in this system. The RMSD between the simulations coordinate snapshots, and the crystal structure showing the FBG structure at a certain time point deviated from the initial conformation. The system was gradually convergent. The RMSD curves for the whole FBG protein were stabilized with RMSD values around 3 Å after 12 ns simulation. It was observed that the difference in the RMSD values of the entire M-ficolin FBG system was slightly larger at pH 7.4 (Figure S1a). Overall, although it appears that insignificant changes were observed for the whole FBG, subtle conformational shifts in the P subdomain seemed to have occurred under different pH. Compared with the RMSD values of the whole FBG domain, the P subdomain appeared more condensed at pH 7.4 (Figure S1b).

To understand pH-induced conformational changes on the M-ficolin FBG domain, the backbone root mean square fluctuations (RMSF) of each residue in the system was calculated (Figure 2c). M-ficolin FBG showed higher fluctuation at pH 6.5 in almost all the residues except for residues in the N-terminus, suggesting a gain of structural plasticity under infection-inflammation condition (pH 6.5, 2.0 mM Ca^2+^). Significant fluctuations were observed at the C-terminus at both pH values. M-ficolin FBG seems to be more compact at pH 7.4 than at pH 6.5.

The backbone dihedral angles of the protein, which are highly correlated with the secondary structures, provide crucial information about its local three-dimensional structure. The occurrence of the backbone dihedral angle, ω (*omega*, involving the backbone atoms C^α^-C'-N-C^α^) of Asp282-Cys283, was plotted (Figure 2d). At each pH, 20000 snapshots from a whole simulation were analyzed. The typical *trans* conformation of the peptide bond usually restricts ω to ±180° and the *cis* conformation restricts ω to 0°. In our work, the initial structure is in neutral condition with *cis*-Asp282-Cys283 bond. We found that at pH 6.5 ω is around 0° during the whole simulation process, which shows that only *cis*-Asp282-Cys283 bond exists. However, two sets of ω values were found around ± 180° and 0° at pH 7.4. The results indicate that at pH 6.5, only the *cis* isomer exists and at pH 7.4, the *trans* isomer appears. Conceivably, this difference in the pH-mediated *cis-trans* populations accounts for the marked change in M-ficolin FBG**:**GlcNAc binding.

### pH regulated *cis-trans* isomerization of Asp282-Cys283 peptide bond in WT FBG

**Figure 3:**
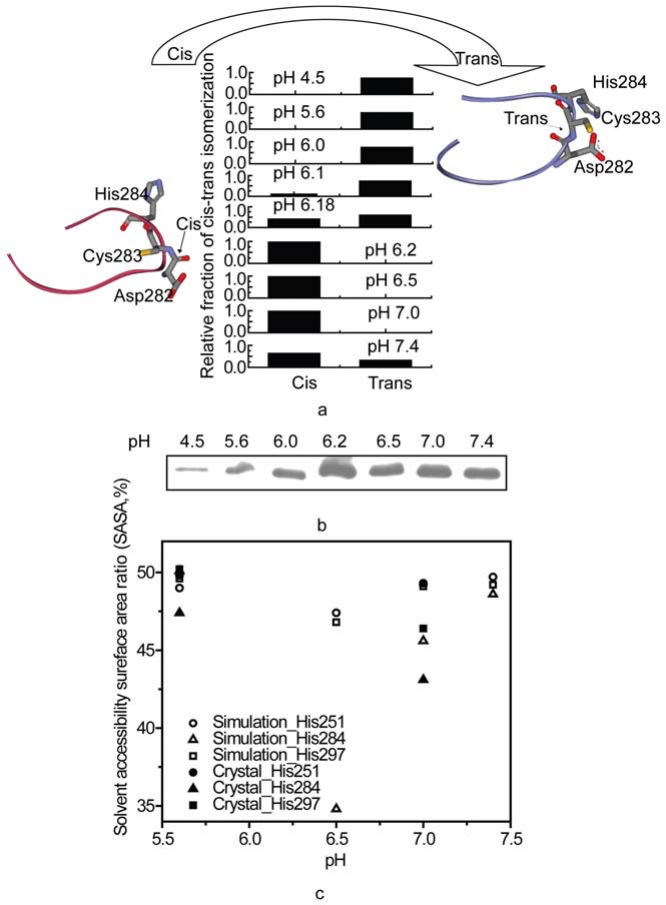
The pH-dependent profile and solvent accessibility surface area (SASA) ratio of M-ficolin FBG domain. (a) *cis-trans* isomerization of Asp282-Cys283 peptide bond. As controls, the crystal structure of M-ficolin FBG domain at pH 7.0 was used as the starting structure of all pH constant simulations. The total simulation data were analyzed. The relative fractions between *cis* and *trans* conformations are shown at pH 4.5, 5.0, 6, 6.1, 6.18, 6.2, 6.5, 7.0 and 7.4. (b) GlcNAc binding activity of the wild type M-ficolin FBG at room temperature by immunodetection analysis. (c) SASA ratio of selected residues at selected pH.

The studies of the M-ficolin-GlcNAc binding activity were carried out to clarify the biological significance of pH-dependency [6], [20], [21], [23], [28], which suggested that there were 2-state conformations at different pH environment. The constant pH simulation results were consistent with the reported data [6] (Table 2). The Asp282-Cys283 peptide bond was dominant with *trans* conformation at pH 4.5, 5.6, 6.0, 6.1 and 6.18, but *cis*-conformation at pH 6.2, 6.5, 7.0 and 7.4. Interestingly, at pH 6.1, 6.18 and 7.4, the competition between the two *cis-trans* isomers was observed, suggesting the gradual titration of the protein groups. Our simulation results showed that the *cis*-conformation of Asp282-Cys283 peptide bond is pH-sensitive (Figure 3a). Therefore, different pH affects not only the stability of the loop regions but also the *cis-trans* conformation of the Asp282-Cys283 peptide bond, and this might lead to the different activities of FBG at different pH environment. This gradual transition between the two isomers at different pH indicates that the transition is associated with the deprotonation of titratable groups. This result is in good agreement with the experimental immunodetection of M-ficolin binding to GlcNAc-beads, which indicated the most intense band at pH 6.2 (Figure 3b). The low pH-induced peptide bond in *trans* showed substantially lesser binding activity for GlcNAc, whereas the peptide bond in *cis*, starting from pH 6.2 and above, showed higher binding activity.

**Table 2:** Dominant conformation (*cis*/*trans*) of the Asp282-Cys283 peptide bond of wild type (WT) crystal structures and constant pH simulations (WT and Mutants).

Structure	Peptide Bond of Asp282-Cys283	pH 4.5	pH 5.6	pH 6.0	pH 6.2	pH 6.5	pH 7.0	pH 7.4
Crystal	WT	*-*	*trans*	*-*	*-*	*-*	*cis*	*-*
	WT	*trans*	*trans*	*trans*	*cis*	*cis*	*cis*	*cis*
	Mutant H251A	*-*	*-*	*-*	*-*	*cis*	*-*	*cis*
Simulation of protein with all groups titrated	Mutant H284A	*-*	*-*	*-*	*-*	*trans*	*-*	*trans*
	Mutant H297A	*-*	*-*	*-*	*-*	*cis*	*-*	*trans*
Simulation of protein with His284 fixed neutralized	WT	*-*	*-*	*-*	*-*	*-*	*cis*	*-*
Simulation of protein with His284 fixed protonated	WT	*-*	*-*	*-*	*-*	*-*	*trans*	*-*

To explain the site-dependent GlcNAc binding activity of M-ficolin, which was earlier observed [28], we further investigated the difference in binding of GlcNAc to the His residues (His251, His284 and His297) by molecular simulation at the atomic level. We analyzed the pH-regulated solvent accessible surface area of His residues in the P subdomain (Figure 3c). His284 was buried within the protein at neutral pH 7.0, but was exposed to the solvent at pH 5.6. It is consistent with the reported crystal structure [6], [23], [25]. The solvent accessibility of His251 located at the beginning of the P subdomain varied slightly at different pH. The three His residues were more exposed at pH 7.4 than pH 6.2 and 6.5, which were the binding-active pH values. His284 is located at the binding site of GlcNAc, and the solvent accessible surface area (SASA) ratio of His284 was small at pH 6.5, which presumably maintains the hydrophobic pocket and *cis* form of Asp282-Cys283 for GlcNAc. This indicates that a conformational change involving His in the M-ficolin would affect the pK_a_ values, thereby changing the active and non-active conformations. The considerable packing difference suggests that His284 side chains may be immobilized at neutral pH but are dynamic at low pH.

### Influence of Histidine on pH regulated *cis*-*trans* isomerization of Asp282-Cys283 peptide bond

**Figure 4:**
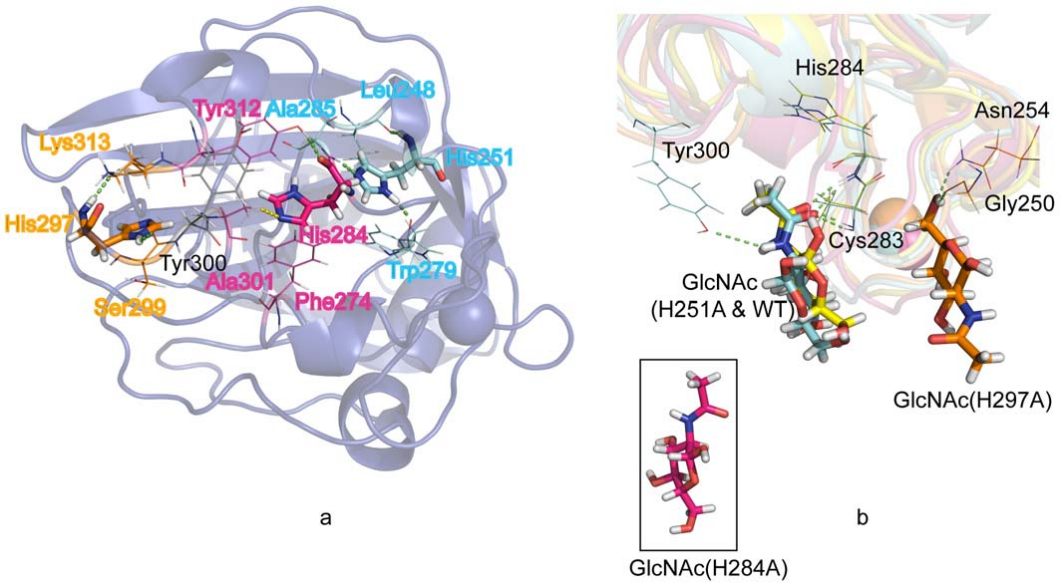
The overall simulated M-ficolin FBG mutants with GlcNAc superimposition. (a) Crystal structure of WT M-ficolin FBG. (b) Centroid simulation structure of WT, H251A, H284A and H297A mutants. The crystal structure in (a) is in grey and the simulated WT, H251A, H284A and H297A in (b) are in yellow, cyan, pink and orange, respectively. The yellow dashed line in (a) represents weak hydrogen bond and the green dashed lines in (a & b) show the hydrogen bond. Ca^2+^ is shown as spheres. The RMSD of crystal structure and the simulated centroid structure of WT is 1.074 Å. The inset is the free/non-bound GlcNAc which ‘escaped’ from the H284A mutant. The WT or His mutants to which the GlcNAc is bound or ‘escaped’ from, is indicated in brackets.

The intramolecular hydrogen bonds of the P subdomain histidines are shown in Figure 4a. The backbone NH group of His251 was found to be hydrogen bonded to the carbonyl oxygen of Leu248. Hydrogen bonds were formed between the imidazole ring of His251 and Trp279, Ala285 and Ser286. The carbonyl oxygen of His251 formed a hydrogen bond with Asn254. His284 was stabilized by the hydrogen bond between the carbonyl oxygen and the hydroxyl group of Tyr312. His297 formed hydrogen bonds with the carbonyl oxygen of Lys313 and Ser299 via the backbone NH and the hydrogen on the imidazole ring, respectively.

The switch of the Asp282-Cys283 peptide bond from *cis* to *trans* form at acidic pH was coupled with the disruption of the H-bond network in the GlcNAc binding site (Figure 2b). The histidine residues were fully protonated at acidic pH, breaking up the hydrogen bond between Ala285, Ser286 and His251 at neutral pH, which was suggested to be associated with the displacement of the loop on the surface of M-ficolin FBG. At neutral pH, the greater stability of the *cis* isomer compared to the protonated protein can be explained by a favorable H-bond interaction between the carbonyl oxygen of His284 and the hydroxyl group of Tyr312 (Figure 4a). The neutral His284 is indeed more tightly H-bonded with Tyr312 than the protonated His284 (54% H-bond population versus 9.4%, respectively) in the first nanosecond simulation. The weak H-bond between the imidazole group of His284 and the hydrogen in the methyl group of Ala301 also helped to stabilize the His284. The protonated His284 caused steric hindrance to Phe274 and Ala301 as well as the loss of the hydrogen bond between His284 and Tyr312 (Figure 4), which led to the reorientation of His284. Additionally, the backbone dihedral angle, ϕ (phi, involving the backbone atoms C'-N-C^α^-C') of Cys283-His284 underwent a shift from ∼−160° to +40° or −60°, indicating the side chain motion. The protonated His284 was spatially closer to Asp282-Cys283 (Figure 2b), and it occupied the binding site of GlcNAc, which is consistent with the crystal structure at pH 5.6 [6]. In order to further clarify the role of His284 in the *cis*-*trans* isomerization of the Asp282-Cys283 peptide bond, we compared the different effects of the protonation states of His284 at pH 7.0 (Table 2). His284 was adopted as fixed neutralized, fixed protonated and titratable states, at pH 7.0. The *cis* conformation of Asp282-Cys283 peptide bond was maintained in the fixed neutralized and titratable His284 states. However, the peptide bond was consistently changed to *trans* even at pH 7.0 when His284 was in the fixed protonated state. Therefore, we propose that the *cis*-*trans* isomerization of Asp282-Cys283 peptide bond is an important indicator of the M-ficolin:GlcNAc binding activity, being mainly regulated by the His284 protonation states in weakly acidic-to-neutral condition.

### Distinctive differences in GlcNAc binding to His-mutants of FBG

#### (a) His-mutants show loss or change in hydrogen bonds with GlcNAc

To further explain the role of the three His residues in binding the GlcNAc ligand at the atomic level, we attempted traditional MD simulation in explicit solvent for the WT FBG with GlcNAc and the His-mutant FBGs with GlcNAc. For the WT, the centroid structure agreed well with the crystal structure, showing an RMSD value of 1.074 Å for all atoms.

**Figure 5:**
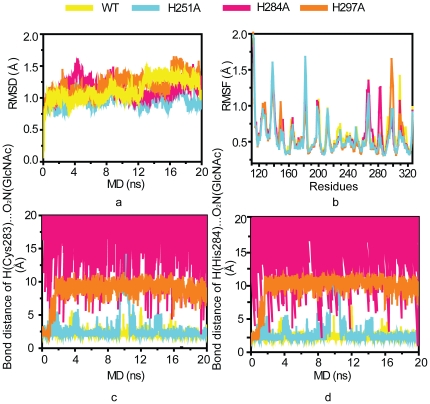
Simulation analysis of WT M-ficolin FBG domain and mutants. (a) Time-dependent RMSD for M-ficolin FBG domain WT, H251A, H284A and H297A. (b) RMSF for M-ficolin FBG domain WT, H251A, H284A and H297A. (c) Hydrogen Bond distance between backbone hydrogen of Cys283 and carboxyl oxygen of GlcNAc. (d) Hydrogen Bond distance between backbone hydrogen of His284 and carboxyl oxygen of GlcNAc. The WT, H251A, H284A and H297A were labeled yellow, cyan, pink and orange and plots were "smoothed" by setting the sliding window average to 10.

In the native state, GlcNAc mainly formed hydrogen bonds with Cys283 and His284 through the carboxyl oxygen. The hydroxyl group of Tyr300 also contributed a hydrogen bond with NH group of GlcNAc to stabilize the complex (Figure 4b). In order to study the effects of the histidine residues, we performed MD simulations of the mutants of M-ficolin FBG (H251A, H284A and H297A) and similar RMSD values were observed, indicating the change of local conformation (Figure 5a), which affected the overall GlcNAc binding. The RMSF (Figure 5b) showed that higher flexibility was observed at the mutation site of H284A and H297A compared with WT, respectively. H251A represented a similar RMSF pattern as WT. The binding patterns of H251A, H284A and H279A mutants to GlcNAc were intrinsically different from each other (Figure 4b). The H251A mutant shared the same GlcNAc-binding site as the WT during most of the simulation time. The H284A mutant completely released the GlcNAc. The H297A mutant bound GlcNAc to another site, which is different from the native binding site in the WT. To estimate the stability of the M-ficolin**:**GlcNAc complex, the occupancy of all possible hydrogen bonds for each adduct was measured by calculating the percentage of time that hydrogen bonds existed during the simulation. We investigated the hydrogen bonds between GlcNAc and the adjacent residues. The inter-hydrogen bond occupancy (Table 3) shows one three center hydrogen bond with significantly high occupancy between the GlcNAc ligand and the WT FBG molecule. The hydrogen bonds occurred between the carboxyl oxygen (O2N) at GlcNAc and the backbone hydrogen atoms of NH at Cys283 and His284 in the protein.

**Table 3:** Occupancy of hydrogen bonds between GlcNAc and nearby residues on M-ficolin FBG.

		Frequency (%)							
		Wild Type		Mutant H251A		Mutant H284A		Mutant H297A	
Hydrogen bond type	Crystal of M-ficolin FBG	First 5 ns	Last 5 ns	First 5 ns	Last 5 ns	First 5 ns	Last 5 ns	First 5 ns	Last 5 ns
H6O@GlcNAC:O@Gly250	No	-	-	-	-	-	-	-	89.36
O6@GlcNAC:HD21@Asn254	No	-	-	-	-	-	-	-	50.04
H4O@GlcNAC:OD1@Asp282	No	-	-	5.04	10.12	-	-	-	-
H3O@GlcNAC:OD1@Asp282	No	-	-	-	7.24	-	-	-	
H4O@GlcNAC:OD2@Asp282	No	-	-	9.68	14.36	-	-	-	-
H3O@GlcNAC:OD2@Asp282	No	-	-	8.96	14.20	-	-	-	-
O2N@GlcNAC:H@Cys283	Yes	81.88	97.40	64.12	66.24	-	-	16.48	-
O2N@GlcNAC:H@His284	Yes	82.32	88.76	45.24	38.65	-	-	15.52	-
H2N@GlcNAC:OH@TYR300	Yes	19.44	16.64	39.16	26.32	-	-	17.60	-
O3@GlcNAC:HH@TYR300	No	14.28	-	8.72	-	-	-	-	-

In interacting with WT FBG, the GlcNAc ligand spent much more time in the WT FBG conformations that allowed for the hydrogen bond formation. We found His284 residue to play a dominant role in GlcNAc binding, whereas His251 and His297, which have no direct interactions with GlcNAc, were somewhat less important, but potentially enabling. Notably, the whole GlcNAc ligand escaped from the H284A mutant, and all the intermolecular hydrogen bonds were disrupted (Figure 4b and Table 3). With the H297A mutant, the hydrogen bond formed by His297 and Ser299 was no longer conserved, thus increasing the flexibility of the loop between 293-303, which weakened the interaction between Tyr300 and GlcNAc. The hydrogen bonds with GlcNAc were impaired and a new binding site was formed between Gly250 and Asn254, which appeared to stabilize the complex with GlcNAc, albeit with relatively weaker binding. Compared with the WT, the H251A mutant spent less time to form the hydrogen bond between Cys283, His284 and GlcNAc, but it interacted more strongly with Tyr300. New hydrogen bonds between H4O or H3O of GlcNAc and Asp282 were observed, suggesting the unstable complex, although still preserving the proximal binding site similar to that of the WT FBG. The GlcNAc was released transiently and then returned to the binding site during the simulation (Figure 5c–d).

#### (b) pH regulated cis-trans isomerization of the Asp282-Cys283 peptide bond in His mutants

The constant pH simulations of the His mutants: H251A, H284A and H297A, were also performed at pH 6.5 and 7.4. Interestingly, at these two pH values, the Asp282-Cys283 peptide bond in the H251A mutant was maintained in *cis*, but in the H284A mutant, this peptide bond was in *trans*, indicating that the H251A mutant still retained GlcNAc binding activity, but the H284A mutant has lost its binding activity for GlcNAc (Table 2). In addition, the Asp282-Cys283 peptide bond of H297A showed *cis* at pH 6.5 but *trans* at pH 7.4, which was different from the WT. This suggests that in a weak basic solution, the His297 residue contributes to the *cis-trans* isomerization of Asp282-Cys283 peptide bond, which mediates GlcNAc binding. However, it was reported that His297 appeared less effective compared to His284 [28]. Thus, the mutational analyses of the His residues in the M-ficolin FBG domain, under constant pH MD simulation revealed that His251, His284 and His297 each elicits distinctive differences on the influence of GlcNAc binding activity to M-ficolin FBG.

## Materials and Methods

### Binding of M-ficolin FBG to GlcNAc beads at different pH condition

M-ficolin FBG protein with myc/His tag was recombinantly expressed and purified as described previously [20]. TBS (25 mM Tris, 145 mM NaCl) adjusted to pH 7.0, 7.4 and MBS (25 mM MES, 145 mM NaCl) adjusted to pH 4.5, 5.6, 6.0, 6.2, 6.5 and 7.4, respectively, with 1% BSA were used to study the effect of pH on the binding of FBG to GlcNAc. 500 µl of 1 µg/ml FBG was dissolved in the abovementioned buffers and incubated with 30 ml of GlcNAc-BSA beads (Sigma-Aldrich, St. Louis, Mo) at room temperature for 2 h. After three washes with the buffer, the beads were boiled in SDS loading buffer at 95^o^C for 5 min before electrophoresis.

### SDS-PAGE and Western blot

The extracted protein samples were electrophoresed on 12% SDS-PAGE and transferred to PVDF membrane (BioRad, Hercules, CA) for 1 h at 70 volts. The membrane was blocked for 2 h with 3% (w/v) skimmed milk in 50 mM Tris, pH 7.4, 145 mM NaCl, 0.05% (v/v) Tween-20 (TBST). The membrane was probed with anti-myc (1∶3000). After four repeated washes with TBST, the membrane was probed with the corresponding secondary antibody (anti-mouse, 1∶2000) in the recommended titer for 1 h, and washed four times in TBST. The immunosignals were detected using Supersignal West Pico Chemiluminescent Substrate (Pierce, Rockford, IL) and exposed to X-ray film (Fujifilm, Tokyo, Japan).

### System setup

The model of WT M-ficolin FBG was from the crystal structures of GlcNAc-bound M-ficolin (PDB entry code: 2JHK) and the ligand-free M-ficolin (PDB entry code: 2JHM), which were obtained at pH 7.0 with the same residue number [6], [26]. In the present study, we used the residue number for the precursor M-ficolin, and every residue number is thus shifted by 29, from that of the mature protein in PDB database (e.g. His284 corresponds to His255 in the mature protein). These models were used as the template in the following simulations. The mutant models (H251A, H284A and H297A) were built from the native models by replacing target residues with the desired amino acid using Discovery Studio 2.5 (Accelrys Inc.). All hydrogen atoms were added using tleap tool from AMBER 10 package [33]. For the GlcNAc, we employed GLYCAM parameter sets for force field calculations [34]. All MD simulations were performed using SANDER module in AMBER 10 suite of programs. The RMSD and RMSF were calculated using ptraj. The individual conformations for analysis were extracted from the last 5 ns of each MD trajectory. All the trajectories were analyzed for the presence of hydrogen bonds between all possible donors and acceptors based on a distance cut-off of 3 Å between the donor and acceptor, and an angular cut-off of 120° between donor-H-acceptor. All molecular figures were generated using PyMol (http://www.pymol.org).

### Constant pH Molecular Dynamics (MD) simulation in implicit solvent

Recently, with the development of computational methods to study pH-induced structural changes, several constant pH MD methods were developed and applied [29], [30], [35], [36], [37], [38], [39], [40], [41], [42], [43], [44], [45], [46], [47], [48], [49], [50], [51], [52], [53], [54], [55] to examine the macromolecular structures. Constant pH MD simulations were started using the model of M-ficolin WT without ligand. Each of the constant pH simulations was performed using SANDER module of AMBER 10 [30], [42]. The ff99SB force field was employed. The generalized Born (GB) model was used for solvation. The system was minimized with 1000 steps of steepest descent algorithm followed by 1000 steps of conjugate gradient algorithm. The system was gradually heated to 300 K for 20 ps. In the following equilibration stage, the restraints applied on the protein were gradually reduced to zero from 10 kcal·mol^−1^·Å^−2^ in 100 ps. The Asp, Glu, and His residues were titrated in the simulations. The predicted pKa values were calculated based on Henderson-Hasselbalch titration curves. The N-terminal amine (Ser) and C-terminal (Ala) groups were fixed at protonated state for the N-terminus Ser side chain and deprotonated for the C-terminus. The Monte Carlo step was performed for 2 fs each. Non-titrating residues were fixed at their most probable protonation states. Salt concentration was set at 0.2 M. Each MD simulation run was then continued for at least 20 ns at the modeling conditions of pH 4.5, 5.6, 6.0, 6.1, 6.18, 6.2, 6.5, 7.0, 7.4 and one of the His residues (His251, His284 or His297) was fixed in protonation state individually at pH 5.6 and 7.0. The Langevin thermostat was used to maintain the temperature of our system at 300 K. The lengths of bonds including hydrogen were constrained using SHAKE. The time step was 2 fs. The translational and rotational motion was removed at regular intervals of 1000 fs. The trajectories were saved at every ps. In the MD simulations, the cutoff for non-bonded interactions was 30 Å.

### MD simulation in explicit solvent

The M-ficolin**:**GlcNAc complexes were solvated with the transferable intermolecular potential with three interaction sites in truncated octahedral box and neutralized by adding counter ions. About 37,700 atoms were yielded for each system. MD simulations were initiated using the model of WT, and the His mutants (H251A, H284A and H297A) of the M-ficolin**:**GlcNAc complex. The standard MD simulation was carried out at neutral pH (∼7) with all primary amines protonated, and without proton transfer in the solute. For each system, the strategy of simulation included minimization, heating, equilibrating, and production MD. The system was first energy-minimized by using four consecutive rounds of 2000 steps of the steepest descent algorithm followed by 2000 steps of the conjugate gradient algorithm. During the initial 4000 minimization steps, only the hydrogen atoms and water molecules were allowed to move freely. Then each system was allowed to relax by several subsequent minimizations during which decreasing force constants from 500 to 1 kcal·mol^−1^·Å^−2^ were applied to the system. The harmonic restraints applied to the system were slowly relaxed by the end of the energy minimization step. 10 Å cutoff value was chosen to calculate all non-bonded interactions in the system. After the relaxation, the system was gradually heated from 0 to 300 K in 50 ps, followed by constant temperature equilibration at 300 K. The remaining restraint was then gradually reduced to zero. The constant pressure periodic boundary with an average pressure of 1 atm and isotropic position scaling were used to maintain the pressure with a relaxation time of 2 ps. Langevin thermostat was used to maintain the temperature of our system at 300 K. The lengths of the bonds including hydrogen were constrained using SHAKE. Each simulation was performed for at least 20 ns with the time step of 2 fs.

### Centroid structure

The structure from the simulation with the lowest RMSD compared to the averaged structure was considered as the centroid structure of that simulation since the average structures are not actual structures from the simulation.

### Solvent Accessibility

The Solvent Accessibility, which calculates the solvent accessible surface of protein residues, was performed for selected amino acid residues without water. Solvent accessibility represents SASA in Å^2^ and SASA ratio was calculated by using single His residue SASA as a reference. The SASA ratio is the Residue Solvent Accessibility divided by the residue solvent accessibility of the fully exposed His residue, calculated using an in situ tripeptide Gly-His-Gly model. The SASA ratios of the His residues of the centroid structures were calculated using Discovery studio 2.5.

## Supporting Information

Figure S1 **Molecular simulation of M-ficolin FBG domain at pH** **6.5 and 7.4.** (a) Backbone root mean square deviation (RMSD) from crystal coordinates of residues of M-ficolin FBG domain. (b) Backbone RMSD from crystal coordinates of residues of FBG P subdomain.
(TIF)
