# Supplementary figures and images for: Histidine-Mediated pH-Sensitive Regulation of M-Ficolin:GlcNAc Binding Activity in Innate Immunity Examined by Molecular Dynamics Simulations
Source: PLoS One. 2011 May 5;6(5):e19647. doi: 10.1371/journal.pone.0019647 (PMC3088710; doi:10.1371/journal.pone.0019647)

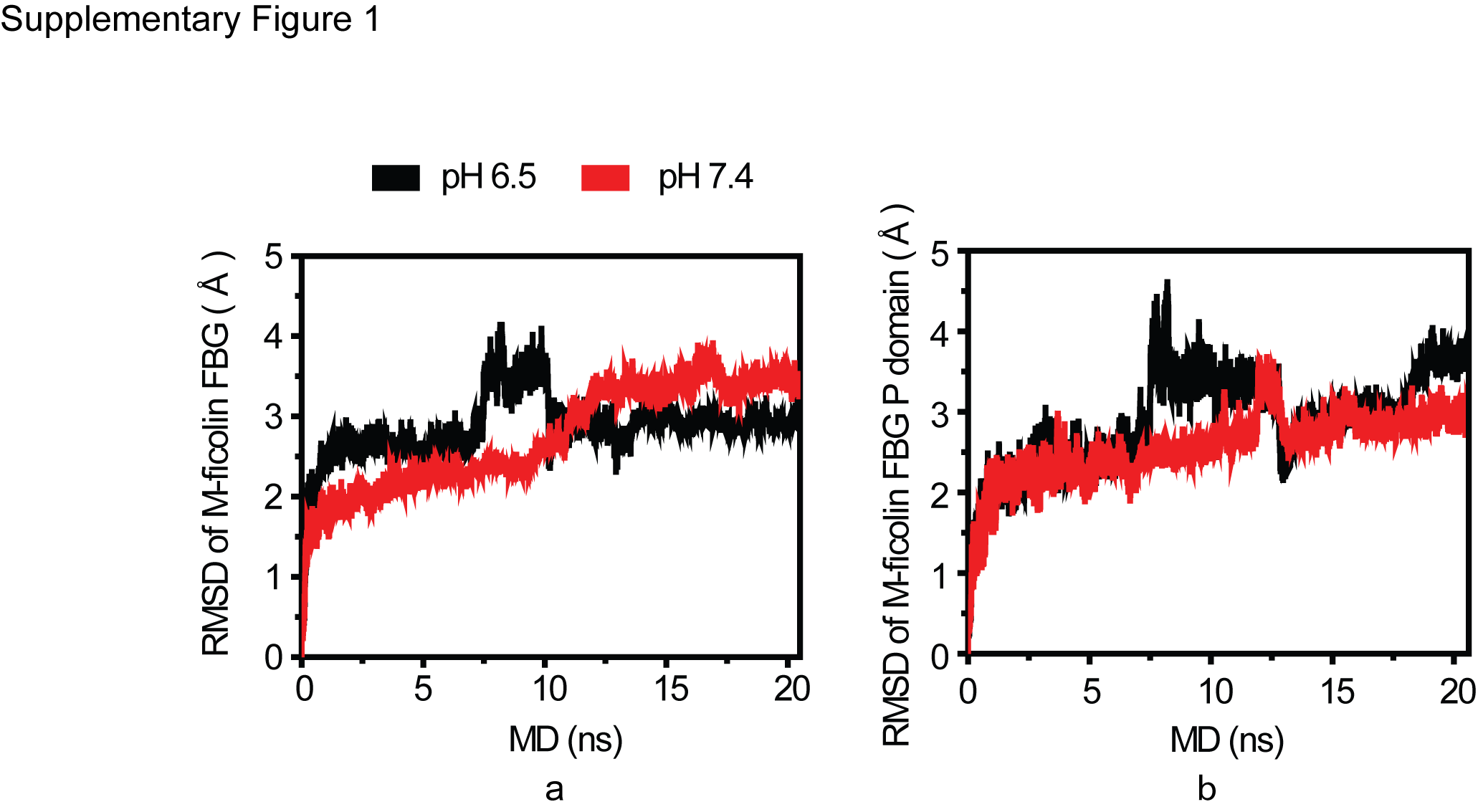

Supplement: Figure S1 — Molecular simulation of M-ficolin FBG domain at pH 6.5 and 7.4. (a) Backbone root mean square deviation (RMSD) from crystal coordinates of residues of M-ficolin FBG domain. (b) Backbone RMSD from crystal coordinates of residues of FBG P subdomain. (TIF) [file pone.0019647.s001.tif]
